# Mechanisms and inhibitors of ferroptosis in psoriasis

**DOI:** 10.3389/fmolb.2022.1019447

**Published:** 2022-09-15

**Authors:** Qiao Zhou, Lijing Yang, Ting Li, Kaiwen Wang, Xiaobo Huang, Jingfen Shi, Yi Wang

**Affiliations:** ^1^ Health Management Center, Sichuan Academy of Medical Science and Sichuan Provincial People’s Hospital, University of Electronic Science and Technology of China, Chengdu, China; ^2^ Department of Rheumatology and Immunology, Sichuan Academy of Medical Science and Sichuan Provincial People’s Hospital, University of Electronic Science and Technology of China, Chengdu, China; ^3^ Clinical Immunology Translational Medicine Key Laboratory of Sichuan Province, Sichuan Provincial People’s Hospital, University of Electronic Science and Technology of China, Chengdu, China; ^4^ School of Medicine, University of Electronic Science and Technology of China, Chengdu, China; ^5^ Department of Rheumatology, Wenjiang District People’s Hospital, Chengdu, China; ^6^ School of Medicine, Faculty of Medicine and Health, The University of Leeds, Leeds, United Kingdom; ^7^ Department of Critical Care Medicine, Sichuan Academy of Medical Science and Sichuan Provincial People’s Hospital, University of Electronic Science and Technology of China, Chengdu, China; ^8^ Department of Rheumatology and Immunology, Sichuan Academy of Medical Science and Sichuan Provincial People’s Hospital, Wenjiang District People’s Hospital, Chengdu, China

**Keywords:** psoriasis, ferroptosis, lipid metabolism, ferrostatin-1, treatment

## Abstract

Psoriasis is a chronic inflammatory skin disease that features localized or widespread erythema, papules, and scaling. It is common worldwide and may be distributed throughout the whole body. The pathogenesis of psoriasis is quite complex and the result of the interplay of genetic, environmental and immune factors. Ferroptosis is an iron-dependent programmed death that is different from cell senescence, apoptosis, pyroptosis and other forms of cell death. Ferroptosis involves three core metabolites, iron, lipids, and reactive oxygen species (ROS), and it is primarily driven by lipid peroxidation. Ferrostatin-1 (Fer-1) is an effective inhibitor of lipid peroxidation that inhibited the changes related to ferroptosis in erastin-treated keratinocytes and blocked inflammatory responses. Therefore, it has a certain effect on the treatment of psoriatic lesions. Although ferroptosis is closely associated with a variety of human diseases, such as inflammatory diseases, no review has focused on ferroptosis in psoriasis. This mini review primarily focused on the pathogenesis of psoriasis, the mechanisms of ferroptosis, the connection between ferroptosis and psoriasis and ferroptosis inhibitors in psoriasis treatment. We discussed recent research advances and perspectives on the relationship between ferroptosis and psoriasis.

## Introduction

Psoriasis (PsO) is a chronic, recurrent, and autoimmune skin disorder caused by multiple risk factors, including genetic and environmental factors (Pezzolo and Naldi, 2019; Kanda et al., 2020; Madden et al., 2020; Reid and Griffiths, 2020). The incidence of psoriasis varies greatly worldwide, with a global prevalence of approximately 2%–4% (Liang et al., 2021) or approximately 125 million people (Armstrong and Read, 2020). According to the clinical features, PsO is divided into four types: vulgaris, arthrogryposis, pustular, and erythroderma (Navarini et al., 2017; Carrasquillo et al., 2020; Lo et al., 2020; Nast et al., 2020). Psoriatic lesions initially appear as red papules. With the development of the disease, the red plaques exhibit various morphologies, such as dot-like, map-like, or covered with thick, silvery-white scales (Raychaudhuri et al., 2014). Keratinocytes play an essential role in psoriasis, and their death amplifies the inflammatory effect. A recent study showed that keratinocytes in psoriatic lesions exhibited lipid peroxidation, which is related to a novel type of cell death, ferroptosis (Wen et al., 2019; Shou et al., 2021).

The Brent Stockwell Laboratory formally defined ferroptosis for the first time in 2012, and it is caused by lipid peroxidation due to the accumulation of reactive oxygen species (ROS) (Dixon et al., 2012). Lipids are organic substances that play critical roles in human tissues. Lipids form the cell membrane and participate in a variety of processes, including cell proliferation and apoptosis, inflammatory conditions, and immune responses (van Meer et al., 2008; Nowowiejska et al., 2021). Iron, lipids and ROS play important roles in cell survival. However, these factors lead to fatal damage when metabolic disorders occur (Liu and Gu, 2022). Ferroptosis is closely related to a variety of human diseases (Jiang et al., 2021), including tumors, septicemia, bacterial, and viral diseases, some neurodegenerative diseases and autoimmune diseases, such as systemic lupus erythematosus, inflammatory bowel diseases, and PsO, which we will discuss briefly in this review (Gunther et al., 2013; Li et al., 2021; Shou et al., 2021).

## Pathogenesis of psoriasis

The pathogenesis of psoriasis is extremely complicated and is currently believed that the development of the disease is caused by the joint action of genetic and environmental factors, which together result in inflammatory cell infiltration, keratinocyte proliferation, and T cell differentiation, etc. (Ogawa and Okada, 2020; Solmaz et al., 2020). The heritability of psoriasis is approximately 66%–90%, which is one of the highest heritability rates of multifactorial genetic diseases (Traks et al., 2019). Environmental factors, such as infection, obesity, nicotine dependence, etc., also induce and aggravate the progression of psoriasis (Madden et al., 2020; Karpinska-Mirecka et al., 2021; Reolid et al., 2021). For example, drip psoriasis is closely related to acute streptococcal infection, which indicates a link between psoriasis and bacterial infection (Madden et al., 2020; Zhou and Yao, 2022). Although the pathogenic etiology of psoriasis is not clear, psoriasis susceptibility genes are associated with immune mechanisms (Vicic et al., 2021; van de Kerkhof, 2022). The innate and adaptive immune systems play crucial roles (Xu and Zhang, 2017), especially CD4 and CD8 cells and the interleukin-23/T helper 17 pathway (Girolomoni et al., 2017; Ly et al., 2019; Iznardo and Puig, 2021). The abnormal activation and migration of specific T cells to the skin leads to the gradual accumulation of inflammatory cells (Xu and Zhang, 2017). Inflammatory cytokines produced by Th1 and Th22 cells, such as tumor necrosis factor (TNF), interleukin (IL)-17, and IL-22, all contribute to the inflammation. With the induction of IL-23, Th17 cells differentiate, proliferate and secrete IL-17, which disrupts the integrity of the skin barrier and induces keratinocyte hyperproliferation (Rendon and Schakel, 2019).

## Mechanisms of ferroptosis

**FIGURE 1:**
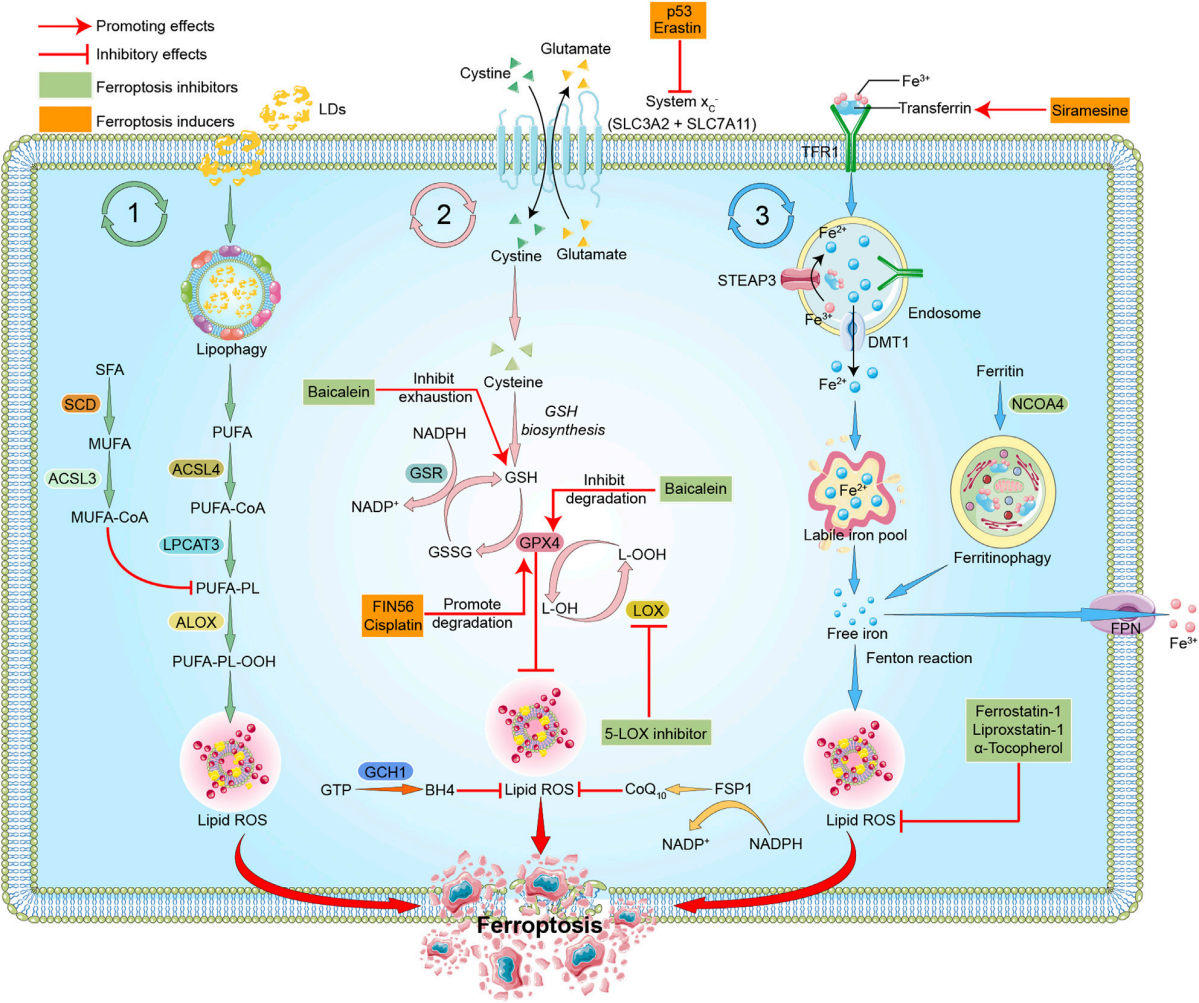
Mechanisms of ferroptosis. The three pathways represent lipid metabolism, antioxidant metabolism, and iron metabolism in ferroptosis, respectively. In lipid metabolism, LDs are degraded *via* lipophagy to release free fatty acids, including PUFAs. Accumulated PUFAs are catalyzed by ACSL4 to generate the key substrate PUFA-CoA, which is finally esterified into PUFA-PLs by LPCAT3. PUFA-PLs can be peroxidized to PUFA-PL-OOH through enzymatic and non-enzymatic lipid peroxidation reactions in the presence of bioactive iron. PLOOH can generate lipid hydroxyl radicals and lipid peroxyl radicals, sensitizing the cell to ferroptosis. MUFAs, likely through MUFA-PLs from MUFA-CoA, can inhibit PUFA-PLs by replacing PUFA from phosphatidylethanolamine, thus reducing the available substrate for lipid peroxidation. In antioxidant metabolism, the two subunits SLC3A2 and SLC7A11 constitute the system Xc-, which is an amino acid antiporter that mediates the exchange of extracellular cystine and intracellular glutamate across the plasma membrane. After entering the cells, cystine is reduced to cysteine and participates in the synthesis of GSH, which serves the substrate of GPX4. GPX4 reduces cytotoxic lipid peroxide (L-OOH) to the corresponding alcohol (L-OH), thus inhibiting the formation of lipid peroxide and ferroptosis. GPX4 can also transform GSH into GSSG, and GSSG can be reduced to GSH under the action of GSR. FSP1-CoQ10 or GCH1-BH4 pathway inhibits ferroptosis independently of GSH. In iron metabolism, ferric iron (Fe^3+^) is bound to transferrin (TF) to form TF-Fe^3+^, which is then taken up by the TF receptor (TFR1). In endosomes, STEAP3 reduces Fe^3+^ to Fe^2+^, which is then released to cytoplasm through DMT1, and stored in LIP or ferritin. Fe^2+^ mediates the Fenton reaction, thereby promoting lipid peroxidation and ferroptosis. Excess Fe^2+^ is oxidized to Fe^3+^ by FPN. In addition, NCOA4-mediated ferritinophagy can increase LIP, thereby sensitizing the cell to ferroptosis through Fenton reaction. The target of ferroptosis inducers and inhibitors are also indicated. P53 and erastin can interfer with the synthesis of GSH by inhibiting system Xc-. FIN56 and cisplatin promote the degradation of GPX4. Siramesine increases the expression of transferrin in iron metabolism and increases the level of intracellular ferric iron. Baicalein inhibits GSH depletion, GPX4 degradation and lipid peroxidation. 5-LOX inhibitor inhibits the production of ROS. Ferrostatin-1, Liproxstatin-1, and α-Tocopherol scavenge ROS and inhibit lipid peroxidation. Red arrows indicate promoting effects. Short lines with vertical end indicate inhibitory effect. The pink ball represents Fe^3+^. Blue balls represent Fe^2+^. Green triangles represent cystine. Yellow triangles represent glutamate. Light green triangles represent cysteine. Green boxes indicate ferroptosis inhibitors. Yellow boxes represent ferroptosis inducers. Abbreviations: ACSL3, acyl-CoA synthetase long chain family member 3; ACSL4, acyl-CoA synthetase long chain family member 4; ALOX, arachidonate lipoxygenase; BH4, tetrahydrobiopterin; CoA, coenzyme A; CoQ10, coenzyme Q10; DMT1, divalent metal transporter 1; FPN, ferroportin; FSP1, ferroptosis suppressor protein 1; GCH1, GTP cyclohydrolase 1; GPX4, glutathione peroxidase 4; GSH, glutathione; GSR, glutathione reductase; GSSG, oxidized glutathione; GTP, guanosine triphosphate; LDs, lipid droplets; LPCAT3, lysophosphatidylcholine acyltransferase 3; LIP, labile iron pool; LOX, lipoxygenase; MUFA, monounsaturated fatty acid; NADP, nicotinamide-adenine-dinucleotide phosphate; NADPH, reduced nicotinamide adenine dinucleotide phosphate; NCOA4, nuclear receptor coactivator 4; OOH, hydroperoxides; PUFA, polyunsaturated fatty acid; PL, phospholipid; ROS, reactive oxygen species; SCD, stearoyl CoA desaturase; SFA, saturated fatty acids; SLC3A2, solute carrier family 3 member 2; SLC7A11, solute carrier family 7 member 11; STEAP3, the six-transmembrane epithelial antigen of prostate 3; TF, transferrin; TFR, transferrin receptor protein.

As a new form of programmed cell death (Dixon et al., 2012), ferroptosis differs from other forms of cell death, such as pyroptosis, apoptosis or cellular senescence, and results in membrane damage and cell lysis (Riegman et al., 2020; Ding et al., 2021). Iron, lipids, and ROS maintain a steady state for cell survival (Liu and Gu, 2022). The accumulation of ROS causes lipid peroxidation and further induces ferroptosis (Stockwell, 2022). The detailed mechanisms of ferroptosis are illustrated below and shown in Figure 1.

### Lipid metabolism

Lipid metabolism is closely related to ferroptosis. As one of the basic elements, long-chain polyunsaturated fatty acids (PUFAs) participate in ferroptosis and are quite sensitive to lipid peroxidation (Li et al., 2020). Free PUFAs are esterified and form film phospholipids that transmit ferroptosis signals after oxidation. PUFAs are linked to coenzyme A (CoA) *via* the catalysis of acyl-CoA synthetase long-chain family member 4 (ACSL4) (Das, 2019). Lysophosphatidylcholine acetyltransferase 3 (LPCAT3) re-esterifies these products into phospholipids and integrates them into membrane phospholipids (Forcina and Dixon, 2019; Li and Li, 2020; Chen et al., 2021b; Liang et al., 2022). Lipid peroxidation leads to the destruction of the lipid layer, which causes cell damage and ferroptosis. Arachidonic acid (AA) is a key phospholipid that induces ferroptosis. ACSL4 and LPCAT3 are involved in the biosynthesis of phosphatidylethanolamine (PE) of adrenaline, a derivative of arachidonic acid, which activates PUFAs and acts as key phospholipids to induce ferroptosis (Stockwell, 2022). ACSL4 and LPCAT3 are also involved in the activation of PUFAs and the binding of them to local membrane lipids, which shows the necessity of PUFAs in the membrane binding environment to have fatal effects on peroxidation (Li et al., 2020; Stockwell, 2022). Because specific carbon atoms in lipids are susceptible to peroxidation, lipid peroxidation depends on the strength of its hydrocarbon bond (Li et al., 2020).

Peroxidation of specific membrane lipids could induce ferroptosis. The latest research shows that cytochrome P450 oxidoreductase is the driver of lipid peroxidation in ferroptosis (Stockwell et al., 2020). Once the production of lipid peroxides far exceeds the antioxidant scavenging capacity, the accumulated lipid peroxides attack the adjacent PUFAs to form new lipid peroxides, which leads to the enhancement of lipid peroxides (Chen et al., 2021a; Stockwell, 2022). Continuous peroxidation of PUFAs leads to changes in the physiological state of cell membranes, such as destruction of the stability and integrity of cell membranes, and ion homeostasis inside and outside of cells (Stockwell, 2022). The decomposition products of lipid peroxide further react with and destroy essential proteins of the human body (Li et al., 2020), which ultimately leads to ferroptosis.

### Iron metabolism

Iron ions are critical factors in the production of ROS *via* enzymatic or non-enzymatic reactions, and it participates in the process of lipid peroxidation and plays a crucial role in ferroptosis (Masaldan et al., 2019; Angelova et al., 2020; Jiang et al., 2021). The abnormal accumulation of free iron in the body often affects normal physiological processes and is a key signal of ferroptosis. Fe^2+^ is acquired from the intestinal absorption or red blood cells degradation. Under the action of ceruloplasmin, Fe^2+^ is oxidized to Fe^3+^, which combines with transferrin (TF) on the cell membrane to form TF-Fe^3+^ complex *via* the membrane protein TF receptor 1 (TFR1) (Frazer and Anderson, 2014). The six-transmembrane epithelial antigen of prostate 3 (STEAP3) reduces Fe^3+^ to Fe^2+^, and Fe^2+^ is normally stored in the labile iron pool (LIP) and ferritin, which is mediated by divalent metal transporter 1 (DMT1) or Zinc-Iron regulatory protein family 8/14 (ZIP8/14) (Li et al., 2020). Excess Fe^2+^ is oxidized to Fe^3+^ by ferroportin (FPN) (Li et al., 2020). LIP is the source of iron ions in the Fenton reaction (He et al., 2020; Lin et al., 2020). Excessive free iron in the cell mediates the Fenton reaction to produce a large amount of ROS, which further causes a cascade reaction, intensifies the lipid oxidation of the cell membrane, and induces ferroptosis (Shen et al., 2018). Ferrous iron may act as a cofactor of some enzymes that mediate lipid peroxidation and participate in the process of ferroptosis. Therefore, the physiological process of iron ions has an impact on the sensitivity of ferroptosis (Xia et al., 2021).

Silencing the TFR1 gene inhibited erastin-induced ferroptosis (Gao et al., 2015). It was also found that heat shock protein β-1 (HSPB1) inhibited ferroptosis by inhibiting TFR1 expression and reducing the intracellular iron concentration (Sun et al., 2015). In contrast, heme oxygenase-1 (HO-1) supplementation with iron accelerated erastin-induced ferroptosis (Kwon et al., 2015).

### Antioxidant metabolism

The antioxidant system is the critical determining factor of the occurrence of iron-induced cell death, and it inhibits the lipid peroxidation chain reaction by reducing lipid peroxides (Seibt et al., 2019). Under physiological conditions, antioxidant enzymes, including glutathione peroxidase 4 (GPX4), inhibit the production of oxidized lipids (Hong et al., 2022). GPX4, which is a glutathione (GSH)- and selenium-dependent glutathione peroxidase, could detoxify lipid hydroperoxides (Miao et al., 2022). GSH is a cysteine-containing tripeptide that exists as an intracellular antioxidant and primarily depends on system Xc-mediated cystine uptake and the concomitant reduction of cystine to cysteine (Ursini and Maiorino, 2020). System Xc- is an amino acid antiporter that is widely distributed in the phospholipid bilayer, and it is a heterodimer composed of two subunits, SLC7A11 and SLC3A2 (Ursini and Maiorino, 2020; Stockwell, 2022). Cysteine and glutamate enter and exit the cell via system Xc-. The absorbed cysteine participates in the synthesis of GSH *via* a reduction reaction. With the help of GSH as a cofactor, GPX4 converts GSH to oxidized glutathione disulfide (GSSG) and reduces cytotoxic lipid peroxide (L-OOH) to the corresponding alcohols (L-OH), thereby detoxifying peroxide products and preventing the accumulation of lipid ROS, making it an important inhibitor of ferroptosis (Friedmann Angeli et al., 2019; Stockwell, 2019; Li et al., 2020; Ursini and Maiorino, 2020; Miao et al., 2022; Stockwell, 2022).

The NADPH-FSP1 (ferroptosis suppressor protein 1)-CoQ_10_ and GCH1 (guanosine triphosphate cyclohydrolase 1)-BH4 (tetrahydrobiopterin) pathways are also reported to be involved in inhibiting ferroptosis, with specific roles needed to be further elucidated (Zheng and Conrad, 2020). RAS-selective lethal 3 (RSL3) and the compounds DPI7 and DPI10 may be used as ferroptosis inducers by directly inhibiting the activity of GPX4, thus reduceing the antioxidant capacity of cells and causing the accumulation of ROS to exert ferroptosis (Yang et al., 2014; Magtanong et al., 2019). P53 downregulates the expression of SLC7A11 and inhibits the uptake of cystine by system Xc-, which may also lead to ferroptosis (Jiang et al., 2015; Liu et al., 2019; Liu and Gu, 2021; Liu and Gu, 2022).

## Ferroptosis in psoriasis

There is an intricate relationship between ferroptosis and inflammation in psoriatic lesions. Abnormal lipid expression and metabolism are frequently observed in patients with psoriasis, especially in keratinocytes from the psoriatic lesions (Benhadou et al., 2019; Shou et al., 2021). At the single-cell level, the lipid oxidation pathway was significantly upregulated in keratinocyte groups of psoriasis, and lipid peroxidation was enhanced during psoriasis (Shou et al., 2021). Compared to other cells, such as fibroblasts, macrophages, dendritic cells, endothelial cells, and T cells, the lipid oxidation activity in keratinocytes highly correlated with the Th22/Th17 pathway and had a time- and concentration-dependent effect on the stimulation of erastin-dependent ferroptosis (Orsmond et al., 2021; Shou et al., 2021).

Cell death related to ferroptosis was also reported to be activated in psoriatic lesions. For example, GPX4 was highly expressed in all layers of the epidermis in normal samples while under-expressed in psoriatic skin, and ACSL4 was highly expressed in the basal layer of the epidermis in psoriasis compared to the normal skin (Shou et al., 2021). The expression of prostaglandin-endoperoxide synthase 2 (PTGS2) and transferrin receptor (TFRC) also increased significantly in psoriatic samples, while the expression of ferritin heavy chain 1 (FTH1) and ferritin light chain (FTL) decreased (Shou et al., 2021). PTGS2 is a potential biomarker for cells undergoing ferroptosis (Wu et al., 2022), and FTH1 and FTL is involved in the storage, entry and homeostasis of ion (Park and Chung, 2019). As a derivative of lipid peroxidation, 4-hydroxynonenol (4-HNE) is also increased in psoriatic lesions and enhances ferroptosis (Liu et al., 2020; Shou et al., 2021). GPX4 is a selenoprotein (Proneth and Conrad, 2019; Zhang et al., 2021b), and the exact reason for its reduced expression in psoriatic lesions is unknown. It is observed that selenium was decreased and related to the severity of psoriasis in patients with long disease duration, and selenium deficiency affects the biosynthesis of GPX4, which might explain the decreased antioxidant activity and susceptibility towards ferroptosis in psoriatic patients (Serwin et al., 2003; Ingold et al., 2018).

Ferroptosis not only promotes cell death, but also triggers inflammation in psoriatic keratinocytes. Several studies showed that ferroptosis triggers and amplifies a variety of inflammatory responses (Tsurusaki et al., 2019; Bebber et al., 2020; Ren et al., 2020). It enhances inflammatory responses *via* the release of damage-associated molecular patterns (DAMPs) and alarmins (Chen et al., 2021a), which could further activate the immune cells and significantly stimulate the expression of inflammatory cytokines, making a complex link between the inflammatory response and ferroptosis in psoriatic lesions.

## Ferroptosis inhibitors in psoriasis treatment

Ferroptosis is involved in several pathophysiological processes and the development of miscellaneous conditions, including iron overload disease and myocardial diseases (Yang et al., 2020; Liu and Gu, 2021; Weber et al., 2021; Lai et al., 2022; Liu and Gu, 2022). Reasonable induction or inhibition of ferroptosis contributes to the treatment of these diseases. For example, the ferroptosis inducer erastin selectively kills tumor cells by regulating oxidative stress (Kose et al., 2019; Zhao et al., 2020). FIN56, siramesine, cisplatin and other inducers also improve the treatment of some diseases (Table 1) (Ma et al., 2016; Guo et al., 2018; Zhao et al., 2020; Zhang et al., 2021a).

**TABLE 1:** The use of ferroptosis inducers and inhibitors in diseases.

Classification		Representatives	Mechanisms	Indications	References
Regulating oxidative stress	Inducers	Erastin	Produce ROS to damage mitochondria or affect GSH synthesis by inhibiting System Xc-	Diffuse large B cell lymphoma	(Kose et al. (2019), Zhao et al. (2020)
		FIN56	Produce ROS and induce ferroptosis by inhibiting GPX4	Glioblastoma	Zhang et al. (2021a)
	Inhibitors	Ferrostatin-1	Inhibit oxidative stress, reduce ROS and lipid peroxidation, and regulate oxidation related proteins such as up regulating GPX4 expression	Psoriasis	Zilka et al. (2017)
		Liproxstatin-1	Reduce mitochondrial ROS production, restore GPX4 level and inhibit lipid peroxidation	Myocardialischaemia/reperfusion	Zilka et al. (2017)
		α-Tocopherol	Damage the chain reaction of automatic oxidation, so as to resist oxidation	Myocardialischaemia/reperfusion	Zilka et al. (2017)
		5-LOX inhibitor	Inhibit glutamate toxicity and ferroptosis by inhibiting the production of ROS in the cytoplasm	Asthma	Yuan et al. (2016)
Iron metabolism	Inducers	Siramesine	Increase the expression of transferrin in iron metabolism and increase the level of intracellular ferric iron	Breast cancer	Ma et al. (2016)
	Inhibitors	Deferoxamine and other iron chelator	Bind free iron ions to inhibit ferroptosis	Thalassemia Major	Guerrero-Hue et al. (2019)
Others	Inducers	Cisplatin	Increase the level of intracellular ROS	Lung cancer	Guo et al. (2018)
	Inhibitors	Baicalein	Inhibit GSH depletion, GPX4 degradation and lipid peroxidation	Pancreatic cancer	Xie et al. (2016)

Abbreviations: GPX4, glutathione peroxidase 4; GSH, glutathione; LOX, lipoxygenase; ROS, reactive oxygen species; System Xc, sodium-independent, anionic amino acid transport system.

However, ferroptosis inhibitors play an essential role, especially in psoriasis. There are many inhibitors, such as ferrostatin-1 (fer-1), liproxstatin-1 (lip-1) and α-Tocopherol (α-TOH) (Xie et al., 2016; Yuan et al., 2016; Zilka et al., 2017; Guerrero-Hue et al., 2019). Fer-1 and lip-1 are more effective compared toα-TOH, which is a relatively weak ferroptosis inhibitor (Stockwell et al., 2017; Shah et al., 2018; Yao et al., 2019; Miotto et al., 2020).

Fer-1 was obtained from the high-throughput screening of a small molecule library. As an aromatic amine antioxidant, Fer-1 prevented erastin-induced lipid ROS production and inhibited ferroptosis in HT-1080 cells, but it did not inhibit the cell death induced by other lethal oxidative compounds, such as H_2_O_2_ or apoptosis inducers (Armstrong and Read, 2020). Fer-1 reduces lipid peroxidation by inhibiting oxidative stress through downregulating prostaglandin endoperoxide synthase 2 and upregulating GPX4 and Nuclear factor E2 related factor 2 (NRF2) (Asano et al., 2017). In psoriatic keratinocytes, Fer-1 blocks the inflammatory responses and alleviates the skin lesions by inhibiting the lipid peroxidation (Kajarabille and Latunde-Dada, 2019; Miotto et al., 2020). Fer-1 was also demonstrated to eliminate erastin-induced death in keratinocytes and alleviate imiquimod-induced psoriasiform dermatitis in mice (Shou et al., 2021). The complex effect of Fer-1 against psoriasis-like inflammatory responses suggests that lipid peroxidation in psoriatic lesions also amplifies inflammatory responses, and the two act together to contribute to ferroptosis (Gong et al., 2019). Further studies need to identify key pathogenic mediators of this process and provide more specific and accurate therapeutic targets.

The level of mammalian targets of rapamycin (mTOR) signaling protein and the expression of mTOR complex 1 (mTORC1) was elevated in psoriatic skin (Raychaudhuri and Raychaudhuri, 2014). Through activating mTORC1, cystine and cysteine promotes the biosynthesis of not only GSH, but also GPX4, while inhibition of mTORC1 sensitizes cells to ferroptosis by decreasing the synthesis of GPX4, demonstrating a link between mTORC1 and ferroptosis (Zhang et al., 2021b; Conlon et al., 2021).

## Conclusion and perspective

Ferroptosis primarily consists of three components: 1) oxidation of PUFAs, 2) excess active iron, and 3) inactivation of GPX4 (Battaglia et al., 2020). Studies have shown that ferroptosis is closely associated with the development of several human diseases, including autoimmune diseases, tumors, infectious diseases, and neurodegenerative diseases, such as Alzheimer’s disease (AD), Parkinson’s disease (PD), and Huntington’s disease (Qiu et al., 2020; Reichert et al., 2020; Lai et al., 2022). The current mini review focused on the mechanisms and treatment of psoriasis associated with ferroptosis and the therapeutic advances that contribute to the effective improvement of psoriatic skin manifestations, including skin thickness and scales, in the role of the inhibition of ferroptosis. Although studies on the role of ferroptosis in psoriasis are scarce, the limited available literature shows that ferroptosis inhibitors have good effects in the treatment of this disease.

However, as a novel cell death mechanism, many issues must be solved urgently. Current research results of ferroptosis in psoriasis are limited, and the specific role of ferroptosis in the occurrence and development of psoriasis is not known. The medical field faces challenges in psoriasis treatment, such as the side effects and adherence of medications. To promote the quality of life of psoriatic patients, we need more effective treatment strategies. Determining the specific connection of ferroptosis and PsO will facilitate targeted therapies and personalized treatment for psoriasis, provide guidance for precision medicine and achieve better therapeutic effects.

Taken together, ferroptosis has a close connection with psoriasis, and there should be more experimental data to support further investigations. Psoriasis treatment strategies based on ferroptosis research will provide great advances in the future and benefit psoriatic patients.
